# Preliminary results of two novel devices for epiphysiodesis in the reduction of excessive predicted final height in tall stature

**DOI:** 10.1186/s10195-022-00666-3

**Published:** 2022-09-17

**Authors:** Andrea Laufer, Gregor Toporowski, Georg Gosheger, Ava von der Heiden, Jan Duedal Rölfing, Adrien Frommer, Anna Rachbauer, Carina Antfang, Robert Rödl, Bjoern Vogt

**Affiliations:** 1 Children’s Orthopaedics, Deformity Reconstruction and Foot Surgery, University Hospital of Muenster, Albert-Schweitzer-Campus 1, 48149 Muenster, Germany; 2 General Orthopaedics and Tumour Orthopaedics, University Hospital of Muenster, Muenster, Germany; 3 Children’s Orthopaedics and Reconstruction, Aarhus University Hospital, Palle Juul-Jensens Boulevard 99, 8200 Aarhus, Denmark

**Keywords:** Temporary epiphysiodesis, Permanent epiphysiodesis, Growth arrest, RigidTack, EpiStop, Tall stature, Height reduction, Knee, Children, Adolescent

## Abstract

**Background:**

In the treatment of tall stature, the reduction of excessive predicted final height can either be achieved by hormonal treatment or surgically by temporary (tED) or permanent (pED) epiphysiodesis. The present study evaluates the preliminary results of two novel devices for tED and pED around the knee to reduce the predicted final height.

**Materials and methods:**

A retrospective analysis was performed to evaluate the clinical and radiographic outcome after bilateral epiphysiodesis for the treatment of tall stature. A cohort of 34 patients (16 girls, 18 boys) who underwent either tED or pED between 2015 and 2020 were eligible for analysis based on the electronic patient records and picture archiving and communication system of our orthopaedic teaching hospital. tED was conducted in 11 patients (32%) through bilateral implantation of four RigidTacks™ (Merete, Berlin, Germany) around the knee. Twenty-three patients (68%) received pED, performed with an EpiStop™ trephine (Eberle, Wurmberg, Germany). The mean overall follow-up time was 2.9 years.

**Results:**

The mean age at surgery was 12.3 years in girls and 13.2 years in boys. Patients had a mean body height of 175.2 cm in girls and 184.7 cm in boys at surgery. The mean predicted final height was 191.4 cm in girls and 210.4 cm in boys. At the last follow-up, 26 patients (76.5%) had achieved skeletal maturity. The mean height of skeletally mature patients was 187.2 cm in girls and 198.5 cm in boys. A mean reduction of the predicted final height of 5.9 cm in girls and 8.7 cm in boys was achieved, corresponding to a reduction in remaining growth of 46% in girls and 38% in boys. Secondary frontal plane deformities of the knee were detected in 5/11 patients (45.5%) in the tED group and 1/23 treatments (4.3%) in the pED group.

**Conclusions:**

tED and pED have both proven to be efficient at achieving growth inhibition to reduce excessive predicted height. However, tED has been associated with an increased risk of secondary angular deformities of the knee. Furthermore, the risk of implant-related complications and the necessity of a subsequent surgical intervention for implant removal have led our study group to abandon tED when treating tall stature. Long-term results of both procedures are pending.

**Level of evidence:**

4.

## Introduction

Tall stature is defined as a height that is more than two standard deviations above the mean for a given age, sex, and ethnic population [1]. While certain endocrine or genetic conditions are known to cause pathognomonic excessive growth, tall stature is most commonly constitutional or familial [2]. Tall stature itself should not be regarded as a disorder, and hence does not require treatment per se [1, 3]. Nevertheless, it may lead to psychosocial impairments; for instance, due to difficulties in finding adequate clothes or a partner [2–6]. In general, surgical options to reduce the final height of a skeletally immature individual should be discouraged. However, if patients suffer psychological strain due to excessive body height and cannot be convinced to refrain from surgical options to reduce the final height, surgical treatment can be considered. Hormonal treatment has been the most commonly applied approach since the mid-twentieth century [2]. However, recent studies have linked the high-dose application of sex steroids with an increased incidence of malignant tumours [7] and reduced fertility [8, 9]. Thus, hormonal treatment of tall stature has considerably declined over the last two decades [2, 7].

A reduction of the predicted final height can also be achieved through surgical procedures targeted at the growth plates around the knee, which induce premature inhibition of the physes of the distal femur and proximal tibia [10]. This procedure may also improve body proportions in patients presenting disproportionally increased leg lengths [10]. Epiphysiodesis can either be performed temporarily (tED) by reversible growth inhibition through devices bridging the physis, or permanently (pED) through definite physeal ablation [10, 11]. Various devices for tED have been established, with varying success and complication rates [12–14]. Recently, a novel rigid staple for tED (RigidTack™, Merete, Berlin, Germany) has been introduced which combines the advantages of staples and plates [11, 15]. pED has historically been conducted according to the techniques originally described by Phemister [16] and Bowen [17]. However, while the Phemister technique is deemed inappropriately invasive, percutaneous drilling is associated with an increased fluoroscopy time and demands precise execution [3]. Our study group has developed a new device (EpiStop™ bone trephine; Eberle, Wurmberg, Germany) for pED, which is a synthesis of the Phemister and percutaneous techniques [18]. Thus far, the results of this novel device have not been evaluated.

Most techniques for tED and pED around the knee have primarily been developed for either hemi-epiphysiodesis to achieve coronal realignment of angular deformities or for the correction of leg length discrepancies (LLD) [13, 14, 19–21]. Data on the application of epiphysiodesis in the treatment of tall stature are scarce [10, 11, 22–24]. The present study evaluated the preliminary results regarding the clinical efficacy and potential complications of two novel devices applied for tED and pED around the knee to reduce excessive predicted final height in individuals with tall stature.

## Materials and methods

Radiographic and clinical data on patients treated for tall stature with two novel devices for epiphysiodesis in a single-centre institution between 2015 and 2019 were retrospectively reviewed. This study was approved by our Institutional Review Board (registration number 2020–319 f-S). The study findings are reported according to the Strengthening the Reporting of Observational Studies in Epidemiology guidelines [25].

The number of patients, predicted final height, proportion index, and underlying conditions were recorded, as well as the patient age at implantation (Table 1). The inclusion criteria were a projected height that was three standard deviations above the sex-specific mean height and open growth plates with a remaining growth potential of more than 5 cm. The exclusion criteria were preceding hormonal treatment, a LLD of more than 1 cm, and clinically significant coronal malalignment of the lower extremities.

**Table 1:** Patient data

Variable	Total cohort			Temporary epiphysiodesis		Permanent epiphysiodesis	
	Both genders	Female	Male	Female	Male	Female	Male
Patients, *n*	34	18	16	4	7	14	9
Aetiology, *n*							
Familial	10	8	2	3	2	5	4
Constitutional	15	9	6	0	0	9	2
Marfan syndrome	9	1	8	1	5	0	3
Mean chronological age at surgery, years (range)	12.7 (8.7–17.8)	12.3 (8.7–15.3)	13.2 (10.1–17.8)	10.5 (8.7–12.8)	12.6 (11.4–13.6)	12.8 (10.6–15.3)	13.7 (10.1–17.8)
Mean bone age at surgery, years (range)	12.7 (8.8–15.2)	12.0 (8.8–14.0)	13.3 (10.0–15.2)	12.0 (8.8–12.5)	13.3 (11.6–15.2)	11.9 (11.0–14.0)	13.0 (10.0–15.2)
Mean height at surgery, cm (range)	179.6 (153.0–200.5)	175.2 (153.0–194.5)	184.7 (170.0–200.5)	167.8 (155.0–180.0)	183.5 (171.0–193.0)	177.3 (153.0–194.5)	185.6 (170.0–200.5)
Mean predicted final height, cm (range)	200.6 (180.0–221.5)	191.8 (180.0–210.0)	210.5 (195.2–221.5)	195.5 (187.5–202.5)	212.4 (200.8–221.5)	190.7 (180.0–210.0)	209.1 (195.2–219.0)
Proportion index (range)	0.96 (0.83–1.12)	0.98 (0.87–1.12)	0.94 (0.83–1.04)	0.97 (0.89–1.04)	0.93 (0.87–0.99)	0.98 (0.87–1.12)	0.94 (0.83–1.04)

Patients and legal guardians were informed about the underlying aetiology and that excessive height was a benign condition. Surgical treatment was generally discouraged. Adolescents nearing skeletal maturity in whom the predicted height reduction amounted to less than 5 cm were advised against surgery. However, if patients and families stated that the tall stature caused psychological strain and credibly assured that the estimated reduction of their final height was likely to improve the patient’s self-perceived body image and mental health, surgery was considered. Informed consent regarding potential benefits and risks was obtained from all patients.

**Fig. 1:**
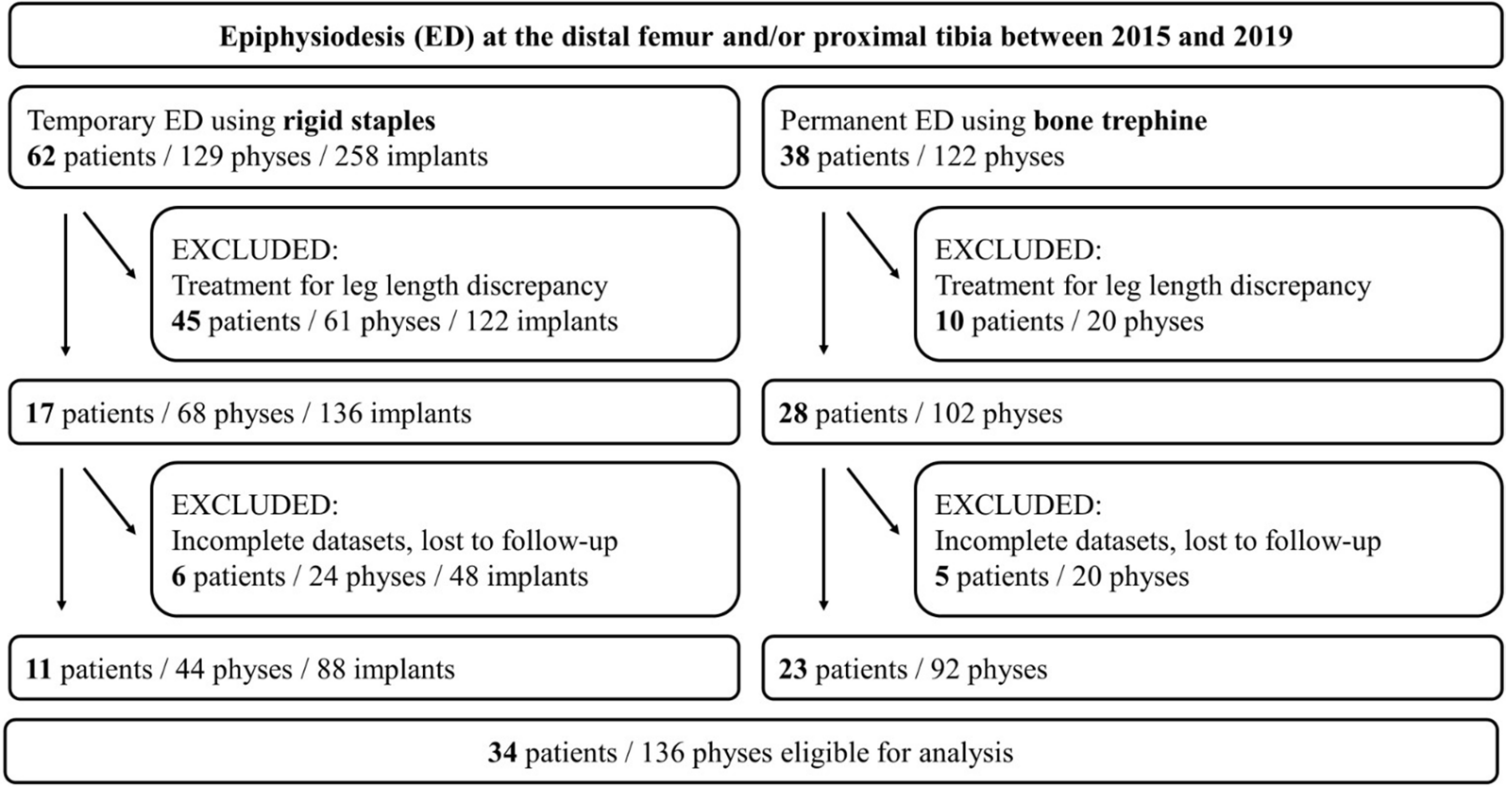
STROBE diagram detailing the inclusion and exclusion criteria of the study

In total, 34 patients (16 girls, 18 boys) with tall stature were included (Fig. 1). tED was performed in 11/34 patients (32%) and pED in 23/34 patients (68%). 14/34 patients (41%) showed familial tall stature, 11/34 patients (32%) presented with constitutional tall stature, and Marfan syndrome had been pre-diagnosed in 9/34 patients (27%). The mean follow-up was 2.9 years (range 0.5–6.0) in the entire cohort, 4.4 years (range 2.0–6.0) in patients treated with tED, and 1.4 years (range 0.5–2.0) in patients treated with pED.

### Prediction of final height and assessment of body proportions

Body height at skeletal maturity was predicted using the multiplier method [26]. Anteroposterior radiographs of the left hand and wrist were obtained to assess bone age according to Greulich and Pyle [27].

Body proportions were evaluated according to Exner, documenting standing and sitting height to evaluate the proportion index [28]. A decreased sitting height/height ratio with a proportion index below 1.0 implies a disproportionally increased leg length of the individual.

### Surgical technique and implants

All procedures were performed by two senior surgeons (RR, BV) who are also authors of this study. Duration of surgery (incision to suture) and fluoroscopy time were recorded.

### Temporary epiphysiodesis

**Fig. 2:**
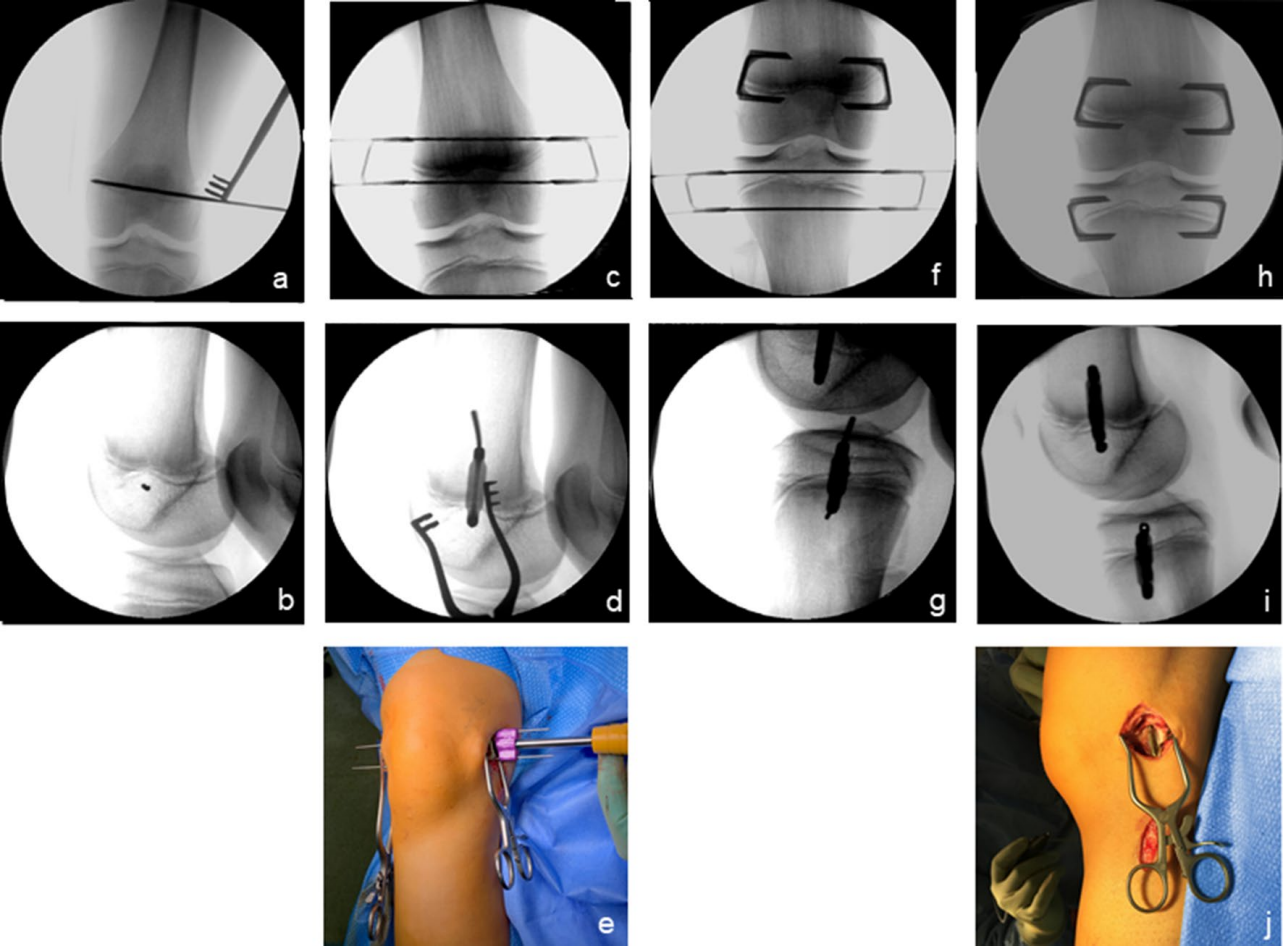
The technique applied for temporary epiphysiodesis using cannulated rigid staples (RigidTacks™). K-wire-guided implantation of opposing rigid staples through medial and lateral incisions at the distal femur (**a**–**e**) and the proximal tibia (**f**–**g**) using a special impact tool. Final result after implantation of rigid staples around the knee (**h**–**j**)

**Fig. 3:**
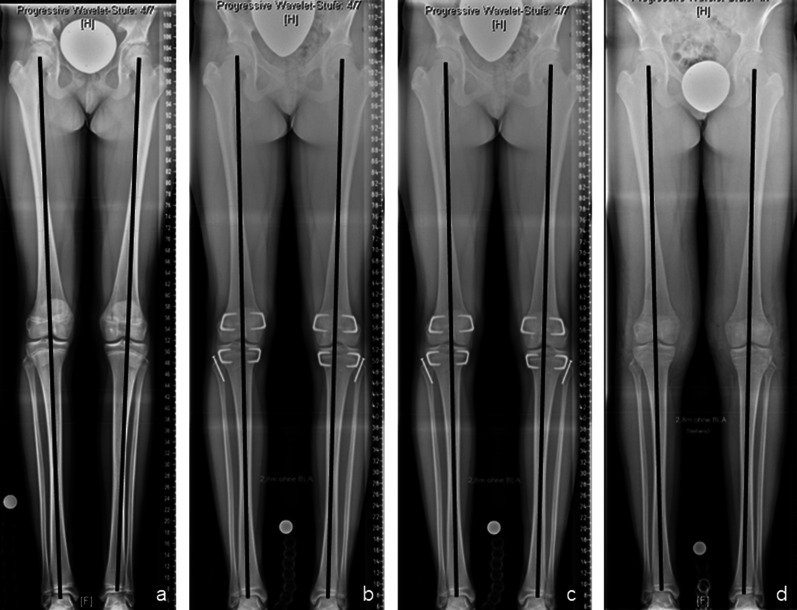
Results of bilateral temporary epiphysiodesis using rigid staples (RigidTacks™) around the knee in a girl with idiopathic (familial) tall stature. Anteroposterior long standing radiographs right before surgery at the age of 12 years (**a**), after 1 year (**b**) and 2 years (**c**) of temporary growth arrest, and right after hardware removal at skeletal maturity (**d**), showing physiological coronal alignment of the knees and equal leg lengths during and after treatment

tED was conducted through the bilateral implantation of four RigidTacks™ around the knee (the lateral and medial distal femurs and the proximal tibia, respectively). This implant provides cannulated barbed prongs for guided insertion and sub-periosteal bone purchase and a reinforced crossbar for sustained compression across the physis during growth. The surgical technique was performed as previously described (Figs. 2, 3) [11].

Implants were retrieved in a subsequent surgery after skeletal maturation.

### Permanent epiphysiodesis

pED was executed using a guided EpiStop™ bone trephine with a diameter of 11 mm. A bone cylinder containing the central part of the growth plate was extracted, and premature fusion of the growth plate was induced through the reimplantation of the cylinder with 90° rotation.

**Fig. 4:**
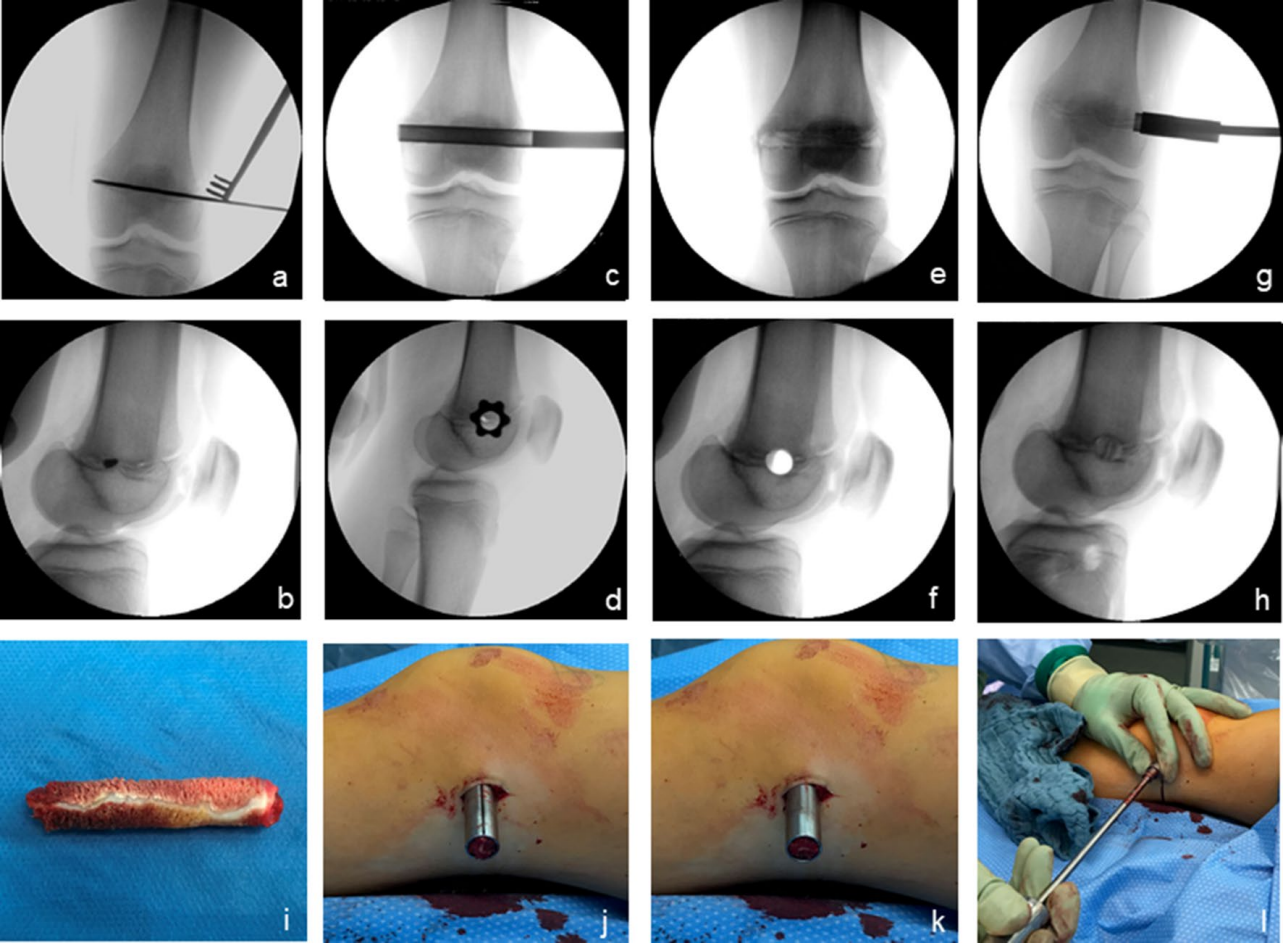
The technique applied for permanent epiphysiodesis using a cannulated bone trephine (EpiStop™). K-wire-guided impaction of the trephine through a lateral incision at the distal femur (**a**–**d**). Fluoroscopic (**e**–**f**) and macroscopic (**i**) control of the complete resection of the central growth plate. Final result after reinsertion of the 90°-rotated bone cylinder, showing the vertically aligned growth plate (**g–﻿h, j–l**)

**Fig. 5:**
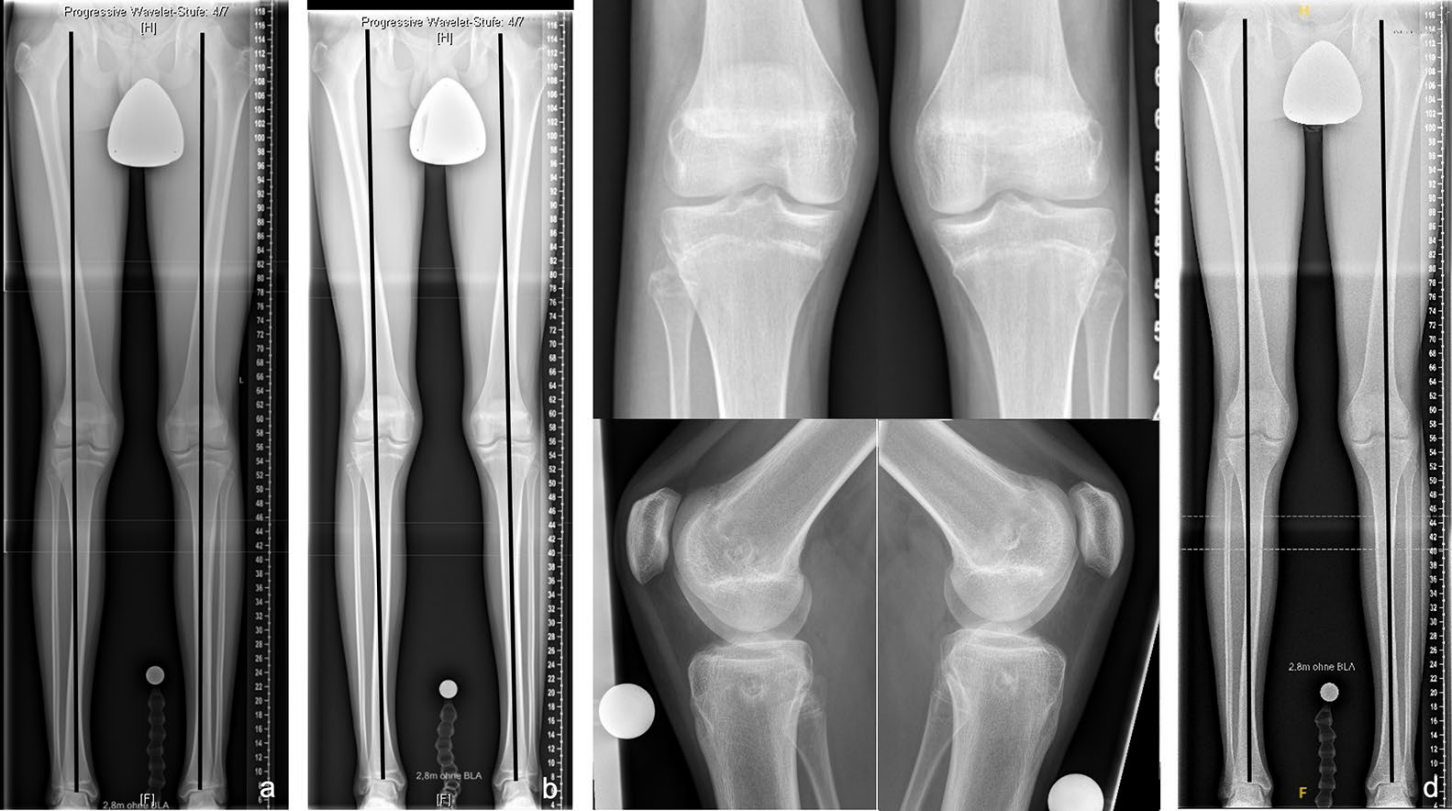
Results of bilateral permanent epiphysiodesis using a bone trephine (EpiStop™) around the knee in a girl with idiopathic (familial) tall stature. Anteroposterior long standing radiographs right before surgery at the age of 13 years (**a**), 6 months after surgery (**b**, **c**), and at skeletal maturity (**d**), showing physiological coronal alignment and equal leg lengths during and after treatment

The procedure was conducted through a lateral incision of approximately 1–2 cm over the distal lateral femur and proximal lateral tibia. A K-wire was inserted through the growth plate, and the correct biplanar positioning in the centre of the growth plate was controlled under fluoroscopy. Subsequently, the trephine was bicortically inserted over the K-wire (Fig. 4a–d). After the retraction of the bone cylinder, complete extraction of the central part of the growth plate was controlled visually and radiologically (Fig. 4e,f,i). Ex situ, the extracted growth plate was rotated 90° under visual guidance before reinsertion. Correct vertical positioning of the reinserted physis was documented with fluoroscopy (Figs. 4, 5).

### Postoperative treatment regimen

All patients were allowed pain-adapted full weight-bearing immediately postoperatively. Physiotherapy was commenced on the first postoperative day and executed daily during the hospital stay and twice per week within outpatient care until full range of motion of the knee joint was regained.

### Clinical and radiological evaluation

Standardised clinical and radiological follow-up examinations at the outpatient department were conducted at 6-month intervals. Standing height and sitting height were recorded using a Harpenden stadiometer. Surgery-related complications were documented.

Radiographs of the left hand were obtained at initial presentation and at the estimated end of growth (approximately 2.5 years after the onset of puberty [29]) to document skeletal maturation. Pre- and postoperative long-standing anteroposterior radiological measurements were obtained. Changes in the mechanical axis (defined as the line between the femoral head and the centre of the tibial plafond) towards varus and valgus malalignment of the lower extremities were recorded. Every shift of the mechanical axis greater than 10 mm was considered clinically important [11]. Measurements of the calibrated radiographs were performed with the PACS^®^ system (GE Healthcare, Chicago, IL, USA) and the post-processing software TraumaCad^®^ (Brainlab, Munich, Germany). In tED, implant placement and bone purchase were reviewed to detect implant-associated complications.

### Statistical analysis

Descriptive statistics were performed using the mean or median with the range. Mean values were compared using the paired *t*-test or the Mann–Whitney *U* test, and dichotomous variables were compared using the chi^2^ test. The level of significance was set at *α* < 0.05. Statistical tests were conducted using SPSS 27 (IBM, Armonk, USA).

## Results

The mean age at the time of the last follow-up was 13.6 years (range 12–16) in girls and 15.8 years (range 14–17) in boys in the tED group, and 14.4 years (range 13–16) in girls and 15.6 years (range 14–19) in boys in the pED group. In the total cohort, the mean age at the last follow-up was 14.9 years (range 12–19), with a mean age of 14.2 years (range 12–16) in girls and 15.7 years (range 14–19) in boys.

Mean height at the time of the last follow-up was 181.0 cm (range 169–187) in girls and 198.8 cm (range 189–205) in boys in the tED group, and 184.3 cm (range 174–200) in girls and 196.6 cm (range 189–201) in boys in the pED group. In the total cohort, the mean height at the last follow-up was 190.0 cm (range 169–205), with a mean height of 183.5 cm (range 169–200) in girls and 197.5 cm (range 189–205) in boys.

The mean proportion index at the time of the last follow-up was 1.04 (range 0.97–1.10) in girls and 0.96 (range 0.85–1.06) in boys in the tED group, and 1.01 (range 0.92–1.11) in girls and 0.96 (range 0.91–1.05) in boys in the pED group. In the total cohort, the mean proportion index at the last follow-up was 1.01 (range 0.85–1.11) in girls and 0.97 (range 0.85–1.06) in boys. Both groups showed a significant improvement in the proportion index at skeletal maturity (*p* < 0.001), with no difference between the groups (*p* = 0.89).

**Fig. 6:**
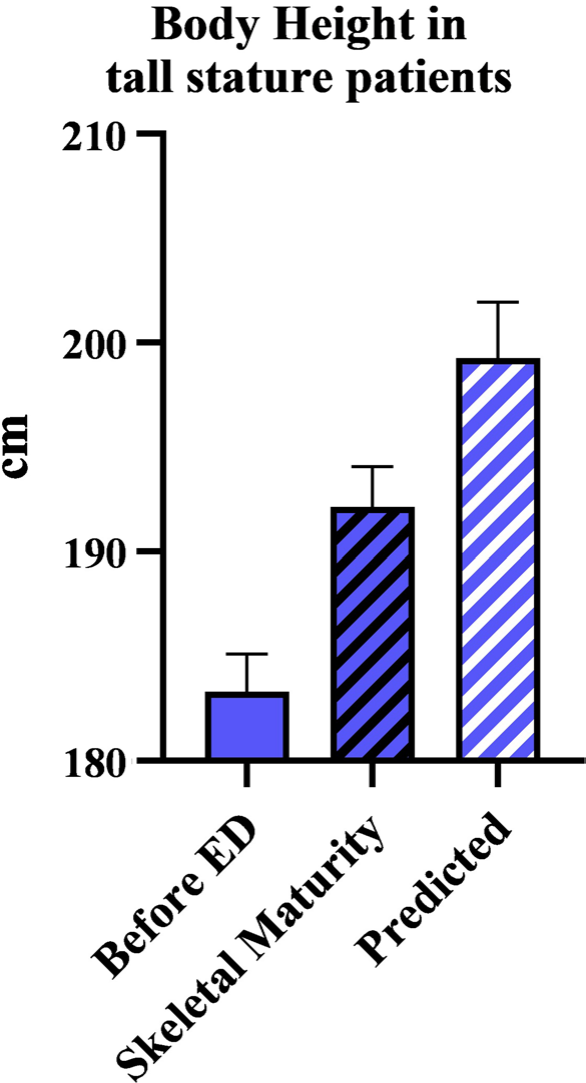
Body height in tall stature patients who were skeletally mature. The plot compares the measured and predicted body heights before epiphysiodesis to the measured body height at skeletal maturity

At the time of the last follow-up, 26/34 patients (76.5%) had achieved skeletal maturity, of whom 9/11 patients (81.8%) were in the tED group and 17/23 patients (73.9%) were in the pED group. The mean height of skeletally mature patients was 184.8 cm (range 183.0–186.5) in girls and 199.8 cm (range 189.0–205.0) in boys in the tED group, and 184.3 cm (range 178.5–190.0) in girls and 199.6 cm (range 194.5–203.0) in boys in the pED group. Overall, the mean height of skeletally mature patients was 187.2 cm (range 183–200) in girls and 198.5 cm (189–205) in boys. Overall, the mean height was 192.7 cm (range 178.5–205.0) (Fig. 6) and the mean proportion index was 0.99 (range 0.85–1.10) in the group of skeletally mature patients at the last follow-up.

Comparing the initially predicted final height with the achieved final height in skeletally mature patients, a reduction of 5.9 cm (range 0–17) in girls and 8.7 cm (range 1–20) in boys was documented, corresponding to a reduction of remaining growth of 46% (range 0–74) in girls and 38% (range 8–61) in boys (Fig. 6). There were no statistically significant differences between the tED and pED groups (*p* = 0.94), with a relative reduction of 41% (range 20–64) achieved by tED and 42% (0–74) by pED. Overall, the reduction of final height proved to be statistically significant in both groups and genders (*p* < 0.001).

Implant removal has been conducted in all six patients in the tED group who have achieved skeletal maturity.

### Chronological age and bone age

**Fig. 7:**
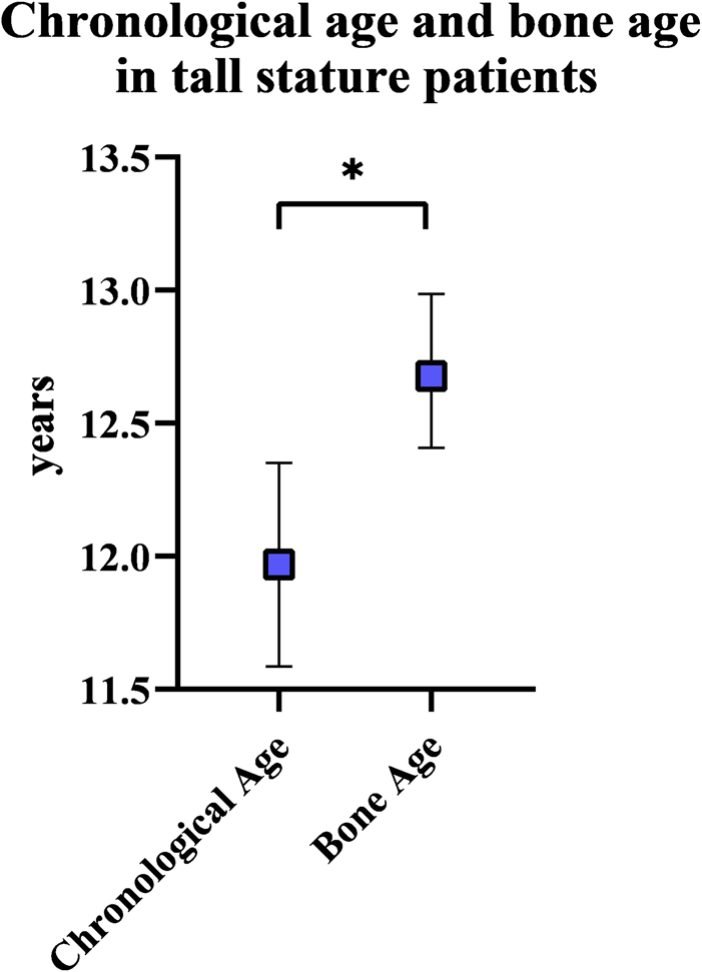
Chronological age and bone age in the entire cohort of tall stature patients at the time of epiphysiodesis

In the entire cohort of 34 tall stature patients, the bone age was significantly advanced by 6.8 months (range − 21 to 36) compared to the chronological age (*p* = 0.020) (Fig. 7).

### Perioperative parameters

Hospitalisation time was 4.5 days (range 3–6) in the tED group and 3.8 days (range 2–7) in the pED group. Overall, patients spent a mean of 4 days (range 2–7) in hospital. The mean time from incision to suture was 166.0 min (range 120–243) in the tED group and 101.5 min (range 60–168) in the pED group. Mean fluoroscopy time was 3.2 min (range 1.03–8.58) in the tED group and 1.7 min (range 0.1–3.64) in the pED group.

### Complications

**Fig. 8:**
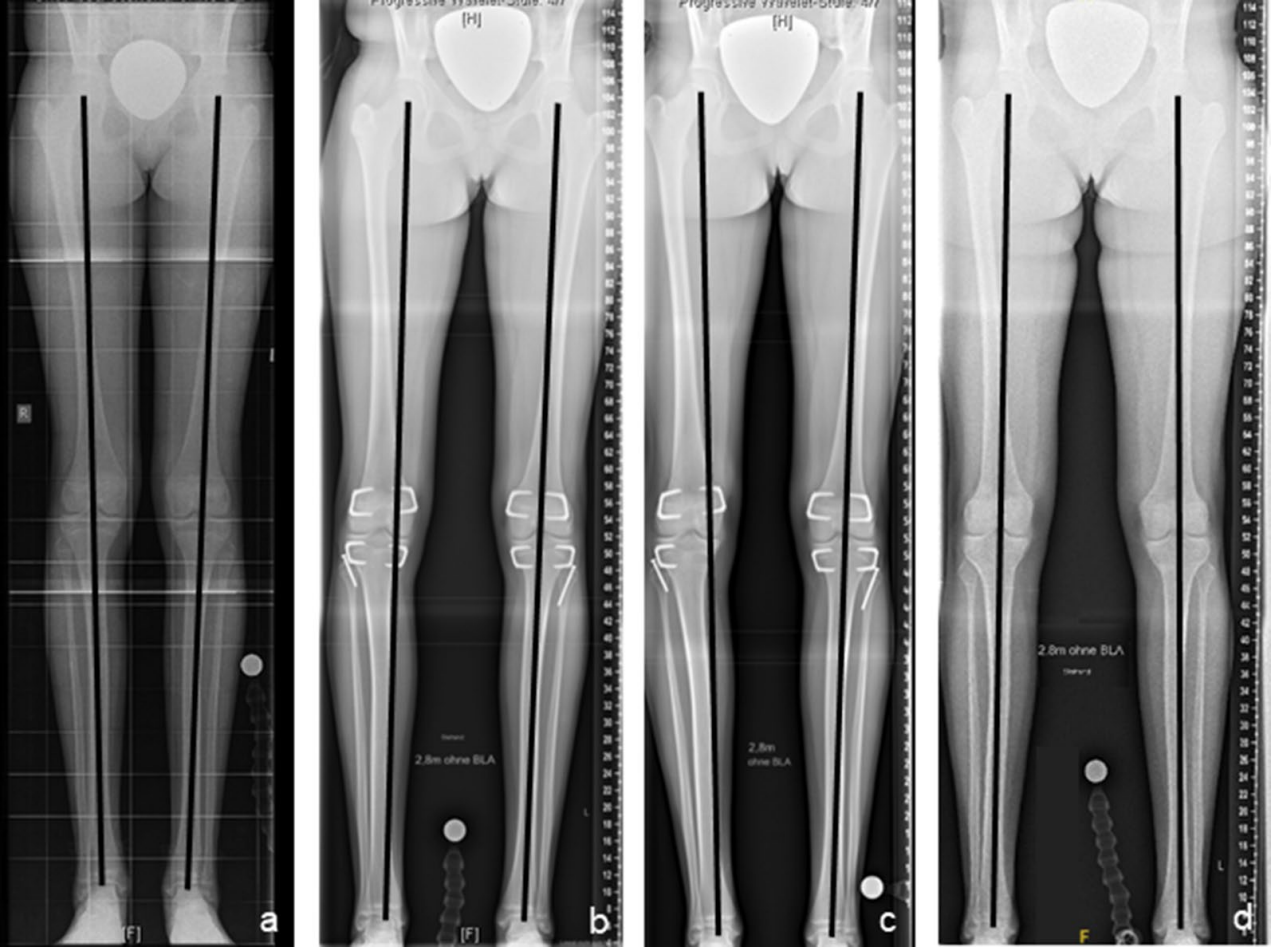
Secondary coronal malalignment after bilateral temporary epiphysiodesis using rigid staples (RigidTacks™) around the knee in a girl with idiopathic (familial) tall stature. Anteroposterior long standing radiographs right before surgery at the age of 10 years (**a**), after 2 years of temporary growth arrest, showing progressive iatrogenic tibial varus deformity on the right side (**b**), right after premature removal of the medial tibial rigid staple at the age of 12 years (**c**), and right after hardware removal at skeletal maturity (**d**), showing physiological coronal realignment and equal leg lengths

Wound infections were observed in 1/11 treatments (9.1%) in the tED group and in 1/23 treatments (4.4%) in the pED group. Neither haematoma, joint effusions, neurovascular complications, nor restrictions of knee joint range of motion occurred in the groups. Implant-associated complications occurred in 1/11 patients (9%) in the tED group. Clinically significant changes of the frontal alignment were detected in 6/22 legs (27%) in 5/11 patients (46%) in the tED group. One was towards valgus and five were towards varus deformity. 4/11 patients (36%) resulted in a mechanical axis deviation (MAD) of ≥ 15 mm at skeletal maturity, but 3/11 patients (27%) had already presented a mild coronal malalignment prior to treatment initiation. The mean change in MAD was 7.0 mm (range 1–31). The patient with valgus deformity was treated by the partial removal of implants at the lateral distal femur and lateral proximal tibia. All patients with varus deformity were treated by implant removal on the concave side of the deformity (Fig. 8). In all affected patients, complete correction of the coronal alignment was achieved.

**Fig. 9:**
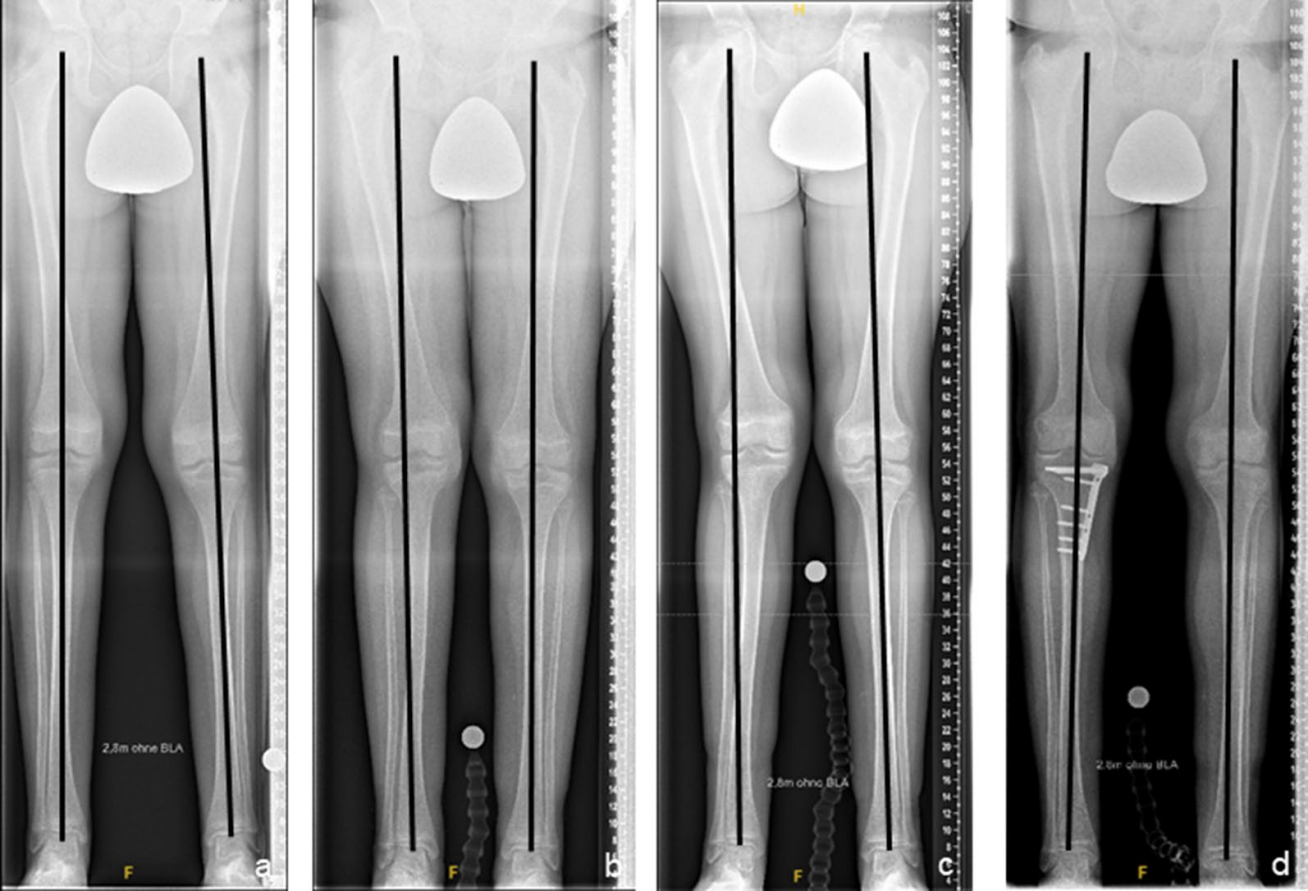
Secondary coronal malalignment after bilateral permanent epiphysiodesis using a bone trephine (EpiStop™) around the knee in a boy with secondary tall stature due to Marfan syndrome. Anteroposterior long standing radiographs right before surgery at the age of 10 years (**a**), 1 year (**b**) and 2 years (**c**) after surgery, showing progressive iatrogenic tibial valgus deformity on the right side, and 2 years after closing wedge correction osteotomy using a locking plate close at the age of 14 years (**d**), showing physiological coronal realignment and equal leg lengths

1/23 patients (4.3%) in the pED group developed a secondary unilateral valgus malalignment with a MAD of − 30 mm and a medial proximal tibial angle of 95°. Coronal realignment was successfully conducted by closing wedge high tibial osteotomy (Fig. 9).

## Discussion

Tall stature has been linked to psychological stress due to perceived psychosocial impairments and a negative body image [4]. However, studies found that reducing the final height through hormonal treatment did not necessarily lead to an improved self-concept and greater patient satisfaction [5, 6]. This highlights the importance of a thorough psychological assessment and psychosocial counselling in young patients requesting treatment for tall stature [6]. Furthermore, hormonal treatment has been associated with severe long-term effects [7–9]. Hence, even though the demand for treatment of tall stature has steadily declined over the past decades—which may be related to an altered perception of tall stature in society—there is a need for alternative treatment procedures [2]. Epiphysiodesis avoids adverse effects associated with systemic therapy. However, despite being minimally invasive, it remains a surgical procedure that is performed in healthy children and adolescents and offers debatable benefits under medical considerations. These concerns should be thoroughly discussed with the patient. Moreover, there should be awareness of the inaccuracy of current methods for predicting final height [2]. This is particularly applicable to patients with primary growth disorders such as Marfan syndrome, for whom—even though specific growth curves exist—validated growth prediction methods have not yet been established [30].

### Efficacy of epiphysiodesis

Both methods of epiphysiodesis investigated in the present study achieved an equally effective reduction of final height. The efficacy of tED and pED appeared to be at least equal or even superior to hormonal treatment. This assumption has also been made by other study groups [2, 10, 22, 23]. However, comparability remains limited, in particular due to differences in cohort sizes, aetiologies, and timing of treatment. Early treatment initiation is crucial to achieve a sufficient growth reduction. In our opinion, assuming that the growth of the femur and tibia cease at around age 14 years in girls and age 16 years in boys, the ideal age for intervention would be 9 years in girls and 11 years in boys, to achieve a growth reduction of about 10 cm. Nonetheless, clinical practice has proven that these calculations are often hard to deploy, since referrals for treatment of tall stature tend to take place rather late.

It appears that individuals with tall stature present an advanced bone age compared to their chronological age, as well as a disproportionately increased leg length [10]. Both assumptions are supported by the findings of the present study. Furthermore, our results indicate that epiphysiodesis may lead to improved body proportions. This observation has also been made by other study groups investigating the effects of epiphysiodesis on stature [10, 22].

Even though they are few, epiphysiodesis implies surgery-related complications which may have a considerable effect on the outcome. In particular, the occurrence of secondary angular deformities may require further treatment to prevent permanent harm. tED offers the advantage of guided growth by converting pan-epiphysiodesis to hemi-epiphysiodesis through partial implant removal on the concave side of the deformity. The occurrence of secondary angular deformities in pED, on the other hand, may necessitate distinctly more extensive surgical procedures, such as corrective osteotomies, if the malalignment exceeds a certain threshold. However, we observed a considerably higher number of secondary axis deviations in the tED than in the pED group. These findings are in accordance with other studies which investigated the efficacy and safety of pED in the treatment of tall stature, where secondary angular deformities were a rare exception [10, 22, 23].

Moreover, in our study, pED with a bone trephine appears to be more cost-effective and was associated with a decreased cut–suture and fluoroscopy time compared to tED. Additionally, tED is associated with implant-related complications and requires subsequent surgery for implant removal. Considering these observations, we have abandoned tED in the treatment of tall stature in favour of pED. Nonetheless, the application of rigid staples in tED for the correction of LLD has shown satisfactory results [11].

## Limitations

This study has several limitations. First, this was a retrospective evaluation of two study groups with heterogeneous cohort sizes, limiting statistical analysis and comparability. Secondly, underlying conditions differed within the patient groups, implying varying growth patterns. Marfan syndrome is associated with a pathological acceleration of growth, leading to limited applicability of common methods for growth prediction, hence diminishing the accuracy in estimating final growth. Thirdly, the age at surgery varied throughout the studied cohort due to the late referral of several patients. The age at which epiphysiodesis is undertaken decisively influences the extent of the height reduction which can be achieved. Since patients within the tED group were younger at the time of surgery, the absolute amount of reduction in final height will inevitably be greater in this group without proving the superiority of the procedure.

Most importantly, not all patients had achieved skeletal maturity at the time of the last follow-up. Long-term results as well as the final height and height reduction will therefore have to be assessed after skeletal maturation of all patients has been achieved, which may influence the study's final outcome.

Nonetheless, our findings support the supposition previously made by other study groups that pED is a safe and effective technique to reliably reduce excessive predicted height in individuals presenting with tall stature [10, 23]. tED, on the other hand, has shown several disadvantages, in particular an increased rate of secondary angular deformities. These findings have led our study group to abandon tED in the treatment of tall stature, yet the procedure is still successfully applied to correct LLD.

## Abbreviations

K-wire: Kirschner wire
MAD: Mechanical axis deviation
n/a: Not applicable
pED: Permanent epiphysiodesis
tED: Temporary epiphysiodesis
